# Phytochemical Variability, In Vitro and In Vivo Biological Investigations, and In Silico Antibacterial Mechanisms of *Mentha piperita* Essential Oils Collected from Two Different Regions in Morocco

**DOI:** 10.3390/foods11213466

**Published:** 2022-11-01

**Authors:** Samiah Hamad Al-Mijalli, Nidal Naceiri Mrabti, Hayat Ouassou, Ryan A. Sheikh, Emad M. Abdallah, Hamza Assaggaf, Saad Bakrim, Mohammed Merae Alshahrani, Ahmed Abdullah Al Awadh, Ahmed Qasem, Ammar Attar, Learn-Han Lee, Abdelhakim Bouyahya, Khang Wen Goh, Long Chiau Ming, Hanae Naceiri Mrabti

**Affiliations:** 1 Department of Biology, College of Sciences, Princess Nourah Bint Abdulrahman University, P.O. Box 84428, Riyadh 11671, Saudi Arabia; 2 Computer Chemistry and Modeling Team, Laboratory of Materials, Modeling and Environmental Engineering (LIMME), Faculty of Sciences Dhar El Mehraz, Sidi Mohamed Ben Abdellah University (USMBA), BP 1796, Atlas, Fez 30000, Morocco; 3 Faculty of Sciences, University Mohammed First, Boulevard Mohamed VI BP 717, Oujda 60000, Morocco; 4 Biochemistry Department, Faculty of Science, King Abdulaziz University, Jeddah 21589, Saudi Arabia; 5 Department of Science Laboratories, College of Science and Arts, Qassim University, Ar Rass 51921, Saudi Arabia; 6 Department of Laboratory Medicine, Faculty of Applied Medical Sciences, Umm Al-Qura University, Makkah 21955, Saudi Arabia; 7 Geo-Bio-Environment Engineering and Innovation Laboratory, Molecular Engineering, Biotechnology and Innovation Team, Polydisciplinary Faculty of Taroudant, Ibn Zohr University, Agadir 80000, Morocco; 8 Department of Clinical Laboratory Sciences, Faculty of Applied Medical Sciences, Najran University, 1988, Najran 61441, Saudi Arabia; 9 Novel Bacteria and Drug Discovery Research Group (NBDD), Microbiome and Bioresource Research Strength (MBRS), Jeffrey Cheah School of Medicine and Health Sciences, Monash University Malaysia, Bandar Sunway 47500, Malaysia; 10 Laboratory of Human Pathologies Biology, Department of Biology, Faculty of Sciences, Mohammed V University in Rabat, Rabat BP 6203, Morocco; 11 Faculty of Data Science and Information Technology, INTI International University, Nilai 71800, Malaysia; 12 Pengiran Anak Puteri Rashidah Sa’adatul Bolkiah Institute of Health Sciences, Universiti Brunei Darussalam, Gadong BE1410, Brunei; 13 Laboratoires TBC, Faculty of Pharmaceutical and Biological Sciences, B.P. 8359006 Lille, France; 14 Laboratory of Pharmacology and Toxicology, Bio Pharmaceutical and Toxicological Analysis Research Team, Faculty of Medicine and Pharmacy, University Mohammed V in Rabat, Rabat BP 6203, Morocco

**Keywords:** *Mentha piperita*, essential oils, phytochemical profile, biological properties, in vitro, in vivo, molecular docking, gas chromatography-mass spectrophotometer

## Abstract

The objective of this work is to explore the phytochemical profile of *Mentha piperita* essential oils (MPEO) collected from two different Moroccan regions using gas chromatography-mass spectrophotometer (GC-MS) and to investigate their antioxidant, anti-inflammatory, antidiabetic and, antimicrobial effects using in vivo and in vitro assays. The chemical constituent of MPEO from the Azrou zone is dominated by carvone (70.25%), while MPEO from the Ouazzane zone is rich in Menthol (43.32%) and Menthone (29.4%). MPEO from Ouezzane showed higher antioxidant activity than EO from Azrou. Nevertheless, EO from Ouezzane considerably inhibited 5-Lipoxygenase (IC_50_ = 11.64 ± 0.02 µg/mL) compared to EO from Azro (IC_50_ = 23.84 ± 0.03 µg/mL). Both EOs from Azrou and Ouazzane inhibited the α-amylase activity in vitro, with IC_50_ values of 131.62 ± 0.01 µg/mL and 91.64 ± 0.03 µg/mL, respectively. The EOs were also tested for minimum inhibitory concentration (MIC) and minimum bactericidal concentration (MBC). The discdiffusion test revealed that MPEOs from both regions have significant antibacterial efficacy, and MPEOs from the north region showed the highest effect. The gram-positive bacteria were the most susceptible organisms. The MIC concentrations were in the range of 0.05 to 6.25 mg/mL, and the MBC concentrations were within 0.05–25.0 mg/mL. The MBC/MIC index indicated that MPEO has strong bactericidal effects.

## 1. Introduction

Man has relied on nature for sustenance and healing since his emergence on Earth. Fossil records show that humans employed the medicinal plant Hollyhock (*Alcea rosea* L.) in Iraq roughly 60,000 years ago, indicating that herbal therapy formed the basis of ancient medicine [1,2]. In the current era, modern pharmaceutical analysis techniques allow researchers to find novel and effective options for treating many related illnesses in the lab. However, creating medications requires a lot of time, money, labor, and specialized tools. Because of this, many people in developing countries now use medicinal plants to treat different diseases [3]. The advent of antibiotics in the 1940s for bacterial infection control revolutionized medicine. These chemicals have successfully restrained microbial infections for decades. However, inexorable bacterial resistance is gradually altering medical practice and threatening the antibiotic era [4]. Recently, over 13 million fatalities worldwide each year are attributed to infectious diseases, despite efforts to prevent and manage them that have been underway for over a century and have had a good deal of success with the aid of antibiotics, which currently fail to control the newly emerging and re-emerging bacterial infectious diseases, in particular [5].

In the literature, numerous prior studies have demonstrated the broad spectrum of antibacterial action shown by medicinal herbs, and due to their efficiency and safety, medicinal plants’ antibacterial properties have paved the way for their use in medical applications [6,7,8,9].

In addition, other pathologies, such as diabetes and chronic inflammation, have a major impact on human health. These two pathologies are linked to several risk factors, in particular microbial infections and oxidative stress [10]. Indeed, the free radicals produced by oxidative stress can damage certain processes, thus leading to dysregulation of blood sugar as well as an inflammatory process downstream.

The management of stress-related pathologies is linked a priori to the prevention of oxidative stress. Antioxidants are natural substances that can inhibit or block free radicals as well as protect against their damaging effects [11].

In this context, natural resources, in particular the molecules contained in medicinal plants, have shown important biological effects, including antioxidant, antidiabetic, and anti-inflammatory activities [12,13]. Among the medicinal plants most used in traditional medicine in Morocco, we find *Mentha Piperita* (*M. piperita*), commonly known as peppermint. It is a genus native to the Mediterranean region that belongs to the Lamiaceae family. The EO of this plant is quite often used in cosmetics, perfumes, food industries, flavors, and pharmaceuticals all around the globe [14].

This plant variety is extensively cultivated for preparing herbal teas and tinctures, or for the management of different conditions and disorders including bowel colic, hepatic disorders, gastritis, headaches, and jaundice [15]. It has shown several biological effects, such as antioxidant and antidiabetic activity [12,16], as well as insecticidal, antibacterial, antiviral, immunomodulatory, antitumor, neuroprotective, antiallergenic, antifatigue, and cytotoxic activities [14,17]. Peppermint as an essential oil for inhalation is effective in relieving symptoms such as vomiting, nausea, and anorexia in patients undergoing chemotherapy [18]. Chemical evaluations revealed that menthol is the principal component of the essential oil, whereas flavone derivatives and cinnamic acid-type phenolic acids were identified as the main compounds of the peppermint extract [19]. The chemical composition and biological effects of this medicinal plant may vary depending on the region and other climatic factors [20,21,22,23,24].

In this sense, the objectives of our work are to evaluate the phytochemical profile of the peppermint EO collected from two different Moroccan regions using GC-MS analysis and to investigate their antioxidant, anti-inflammatory, antidiabetic, and antimicrobial effects using in vivo and in vitro methods.

## 2. Materials and Methods

### 2.1. Reagents

Acarbose, quercetin, ascorbic acid, 2,20-diphenyl-1-picrylhydrazyl (DPPH), 2,2-azinobis-3-ethylbenzothiazoline-6-sulfonic acid (ABTS), 6-hydroxy-2,5,7,8-tetramethylchroman-2-carboxylic acid (Trolox), α amylase, lipase and α glucosidase, allopurinol, and ascorbic acid were purchased from Sigma-Aldrich (Saint-Quentin-Fallavier, France). Lipoxygenase (5-LOX) and linolenic acid were purchased from Sigma-Aldrich (Saint-Louis, MO, USA). Mueller–Hinton Agar, DMSO, and chloramphenicol were purchased from (Biokar, Beauvais, France). All other reagents were of analytical grade.

### 2.2. Plant Material and Extraction

Aerial parts of *M. pepirita* were harvested in June 2021 from the Azrou and Ouazzane regions (Morocco), respectively. The plants were identified according to the procedure described by Tejero et al. [25]. These samples were confirmed at the scientific institute of Rabat, University of Mohammed V Rabat, Morocco, and voucher specimens of each plant were deposited in the herbarium under the voucher specimen codes RAB16822 for the Azrou and RAB13258 for the Ouazzane regions. The samples collected were dried and then stored away from light for a few days before proceeding with the extraction of the essential oils (EOs). EO extraction was carried out by hydro-distillation in a Clevenger-type apparatus (VWR, Radnor, PA, USA). Indeed, 100 g of the dry matter of the aerial part was placed in water and boiled for 3 h. The oil is recovered and then stored at a temperature of 4 °C until further use. 

### 2.3. Determination of Volatile Compounds

The chemical components of *M. piperita* were determined using gas-chromatography/mass-spectrometry (GC/MS) analysis as described previously by Assaggaf et al. [26]. Indeed, a Hewlett-Packard (HP6890) GC instrument (Aligent, Santa Clara, CA, USA) coupled with an HP5973 MS and equipped with a 5% phenylmethyl silicone HP-5MS capillary column (30 m × 0.25 mm × film thickness of 0.25 μm) was used in the GC analysis. The used column temperature increased from 50 °C for 5 min to 200 °C with a 4 °C/min rate. Helium with a 1.5 mL/min flow rate and a split mode (flow: 112 mL/min, ratio: 1/74.7) was the used carrier gas. The hold time was 48 min and the injector and detector were both 250 °C. The machine was led by a computer system of the type “HP ChemStation”, managing the functioning of the machine and allowing us to follow the evolution of chromatographic analyses. Diluted samples (1/20 in methanol) of 1 μL were injected manually. In addition, 70 eV ionization voltage, 230 °C ion source temperature, and a 35–450 (*m*/*z*) scanning range were the MS operating conditions. The quantification of the different compounds was obtained by internal normalization of the total area of peaks detected in each chromatogram. Finally, the identification of different compounds was carried out by the comparison of MS spectra with the library and matching the Kovats index (Library of NIST/EPA/NIH MASS SPECTRAL LIBRARY Version 2.0, 1 July 2002) (National Institute of Standards and Technology (NIST), Gaithersburg, MD, USA).

### 2.4. Antioxidant Activity

#### 2.4.1. DPPH Radical-Scavenging Activity

The essential oil of *Mentha piperita* was performed using a 1,1-diphenyl-2-picryl hydrazyl (DPPH) technique, as reported in Brand-Williams et al. [27] with some changes. Briefly, a 2.5 mL aliquot of DPPH solution was mixed with 1.25 mL of the essential oil at a concentration of 1 mg/mL, and then the obtained solution was incubated at 25 °C for 30 min. The absorbance was measured at 517 nm. Redundant testing was performed in triplicate (*n* = 3), and the percentage of anti-radical activity was expressed as an IC_50_ value (µg/mL).

#### 2.4.2. Trolox Equivalent Antioxidant Capacity (TEAC) Assay

The assay was carried out using the ABTS^+^ method as described by Re et al. [28] with some modifications. Briefly, the ABTS radical cation was generated by mixing 10 mL of 2 mM ABTS with 100 µL of potassium persulfate (2.45 mM). After the reaction mixture is kept in the dark at room temperature for 16 h, methanol is used to dilute the ABTS solution to obtain an absorbance of 0.700 at 734 nm. Two hundred µL of the essential (1 mg/mL) was mixed with 2 mL of diluted ABTS solution. The mixture was allowed to react for 1 min, and the absorbance was measured at 734 nm. Trolox is used as a reference standard. The results were expressed as an IC_50_ value (µg/mL).

#### 2.4.3. H_2_O_2_-Scavenging Activity

The H_2_O_2_ radical scavenging activity of extracts was determined according to the method described by Ruch et al. [29]. Briefly, 40 mM of H_2_O_2_ solution was prepared in phosphate buffer (pH 7.4). A total of 1 mL of *M. piperita* HE at a concentration of 1 mg/mL was added to 0.6 mL of H_2_O_2_ solution. The mixture was incubated at 25 °C for 10 min, and the absorbance of H_2_O_2_ was determined at 230 nm against a blank solution containing phosphate buffer without H_2_O_2_. The positive control used is Ascorbic acid. The H_2_O_2_ trapping capacity was expressed as an IC_50_ value (µg/mL).

#### 2.4.4. Xanthine Inhibition Assay

The xanthine inhibitory activity was determined as previously described by Mohammad et al. [30], with some modifications. The reaction mixture was composed of 1.9 mL of phosphate buffer (pH 7.5), 0.1 mL of enzyme solution (0.2 unit/mL), 1.0 mL of 0.5 mM xanthine solution, and 1.0 mL of *M. piperita* EOs. Subsequently, the enzymatic reaction was stopped with 1mL of 1.0 M HCl. The mixture was incubated for 15 min at 25 °C. The absorbance was measured at 295 nm against a blank solution containing the assay mixture without the enzyme. The positive control is allopurinol. The XO inhibitory activity was expressed as an IC_50_ value (µg/mL).

### 2.5. Antidiabetic Activity

#### 2.5.1. α-Amylase Inhibition Assay

The pancreatic α-amylase inhibition assay was determined according to the modified procedure of Mrabti et al. [31]. Briefly, 200 μL of EO or acarbose (positive control) samples were preincubated with 200 μL of α-amylase solution and 200 μL of 0.02 M phosphate buffer (pH = 6.9) at 25 °C for 10 min. Then, 200 μL of 1% starch solution were added to the reaction mixture. Subsequently, we incubated the melenge at 37 °C for 20 min, then boiled for 5 min after adding 500 μL of dinitrosalicylic acid (DNSA), followed by incubation in a boiling water bath at 100 °C for 8 min to stop the reaction and cool in a water bath. Finally, the reaction mixture was diluted by adding 2 mL of distilled water, and the absorbance was measured at 540 nm. The α-amylase inhibitory activity was expressed as an IC_50_ value (μg/mL).

#### 2.5.2. α-Glucosidase Inhibition Assay

The α-glucosidase inhibitory activity was determined according to the method of Mrabti et al. [31], using α-glucosidase from Saccharomyces cerevisiae. The p-Nitrophenyl-α-D-glucopyranoside (pNPG) substrate is prepared in 100 μL of 0.1 M sodium phosphate buffer (pH = 6.7). After 100 μL of α-glucosidase (0.1 U/mL) were preincubated with 150 μL of EO for 10 min. Then, 200 μL of 1 mM pNPG solution dissolved in 0.1 M sodium phosphate buffer was added to start the reaction. The reaction mixture was incubated at 37 °C for 30 min and quenched by adding 1 mL of 0.1 M Na_2_CO_3_. Absorbance was measured at 405 nm. The α-glucosidase inhibitory activity was expressed as an IC_50_ value (mg/mL).

#### 2.5.3. Lipase Inhibition Assay

Pancreatic lipase activity was determined by measuring *p*-NPB as a substrate. The procedure was modified from that previously described by Kim et al. [32]. A fresh stock solution of pancreatic lipase enzyme was prepared in Tris-HCl buffer (pH = 7.4). A p-NPB stock solution was prepared by dissolving 20.9 mg of p-NPB in 2 mL of acetonitrile. Then, for each working solution, 0.1 mL of porcine pancreatic lipase (1 mg/mL) was added to test tubes containing 0.2 mL of *M. piperita* EO. The resulting mixtures were then made up to 1 mL by adding Tri-HCl buffer and incubated for 15 min at 37 °C. Then 0.1 mL of p-NPB solution was added to each test tube after incubation, and the resulting mixture was again incubated for 30 min at 37 °C. The lipase activity was determined by measuring the hydrolysis of p-NPB to p-nitrophenol at 405 nm using a UV-visible spectrophotometer. The same procedure was repeated for orlistat (a positive control). All established test samples were performed in triplicate. Lipase inhibitory activity was expressed by IC_50_ values.

### 2.6. In Vitro and In Vivo Anti-Inflammatory Activity

#### 2.6.1. Animals

The studies were carried out on adult rats of the Wistar strain, obtained from the central animal facility of the Faculty of Medicine and Pharmacy of Rabat. These rats weighed between 200 and 250 g. The temperature was maintained at 20 °C with a lighting cycle of 12 h of light and 12 h of darkness. The study was approved by the Ethics Committee from Mohammed V University, (Protocol code # UA-2021-09; date of approval 27 May 2021).

#### 2.6.2. In Vitro Anti-Inflammatory Assay

The lipoxygenase inhibitory activity of *M. pepirita* was evaluated by following the linoleic acid oxidation at 234 nm, according to the previous published method [33]. Briefly, 20 µL of oil and 20 µL of Glycine max 5-LOX (100 U/mL) were pre-incubated with 200 µL of phosphate buffer (0.1 M, pH 9) at room temperature for 5 min, with the addition of 20 μL of linolenic acid (4.18 mM in ethanol) to start the reaction. Results are the mean ± SEM of three independent assays, each performed in triplicate. The positive control used is quercetin.

#### 2.6.3. In Vivo Anti-Inflammatory Assay

The anti-inflammatory effects were studied using the carrageenan-induced paw edema method previously described by Musa G. Rege et al. [34]. Briefly, Wistar rats (160 to 200 g) were randomly divided into eight groups (*n* = 6). The first two groups were orally administered with 100 mg/kg of *M. pepirita* EO. The third group was a negative control which received distilled water, while the last group was considered a positive control and received indomethacin (10 mg/kg) as the reference anti-inflammatory drug. After 60 min, all rats were injected subcutaneously with carrageenan solution (0.05 mL of 1% carrageenan suspended in 0.9% NaCl) into the subplantar region of the left hind paw. The paw volumes of the rats were recorded using a LE 7500 digital plethysmometer controlled by SeDaCOM software (Harvard Apparatus, Holliston, MA, USA) just before the carrageenan injection (V0), and then at 1 h, 3 h, and 6 h after the carrageenan injection (Vt). The anti-inflammatory effect is calculated using the following equation. In each group, the anti-inflammatory effect is measured by the percentage of edema inhibition (% INH) in paw volume, as follows:

%INH = (mean [VL − VR] Control − mean [VL − VR] Treated)/[VL − VR] Control × 100

With: VL: volume of the left paw.
VR: volume of the right paw.

### 2.7. Antibacterial Activity

#### 2.7.1. Antibacterial Testing

##### Bacterial Strains

The antibacterial activity of *M. piperita* essential oils (MPEO) from Ouezzane and Azrou regions (Morocco) was evaluated against the following six bacterial strains: three gram-positive bacteria (*Staphylococcus aureus* ATCC 29213, *Listeria monocytogenes* ATCC 13932, *Bacillus subtilis* ATCC 6633), and three gram-negative bacteria (*Escherichia coli* ATCC 25922, *Salmonella typhimurium* ATCC 700408, and *Pseudomonas aeruginosa* ATCC 27853).

##### Inoculum Preparation

An inoculum of the frozen stock (−20 °C) was utilized to inoculate Mueller–Hinton Agar (Biokar, Beauvais, France), where it was incubated for 24 h at 37 °C to revive the bacteria. Then, an inoculum was removed from a bacterial colony and diluted to 0.5 McFarland in sterile salt water (0.9% NaCl) before being transferred to a sterile tube where the weighty particles were left to settle for 5 min. The top homogeneous solutions were transferred to a new, sterile tube and their concentration was adjusted to the 0.5 McFarland turbidity standard to obtain an inoculum of about 10^6^ CFU mL^−1^, which was used in the antibacterial testing

##### Disc Diffusion Method

The disc diffusion approach was selected to determine the preliminary screening of the antibacterial properties of the studied essential oils according to the previously described methodology [35]. Briefly, the culture suspension was inoculated by swabbing on Mueller–Hinton Agar (Biokar, Beauvais, France). Then, 6 mm sterile filter paper discs were placed on each plate and soaked in 10 µL of each essential oil. Chloramphenicol (30 µg) was used as a control. The bacterial plates were incubated at 37 °C for 24 h. After incubation, the inhibitory diameters were measured in millimeters, and results were expressed as means ± standard deviation of three replicates.

##### Minimum Inhibitory Concentration Method

The minimum inhibitory concentration (MIC) is the lowest essential oil concentration that could prevent the growth of bacteria. The determination of MIC values against bacteria was conducted according to the previously reported procedure with a minor change [36], using Mueller–Hinton broth (Biokar, Beauvais, France) and bacteria. Twenty-four h of incubation was done at 37 °C. The antibiotic chloramphenicol served as a positive control. In sterile microtubes, two-fold serial dilutions of EO concentrations ranging from 4% to 0.0625 % (*v*/*v*) were made in Mueller–Hinton broth containing 5% of DMSO. Previous studies showed that dimethyl sulfoxide (DMSO) at a concentration of up to 7.8% (*v*/*v*) % has no noticeable reduction in viable bacterial cell number [37]. Then, 5 µL of the previously produced bacterial suspension was added to each tube. Each suspension was homogenized and incubated for 24 h at 37 °C. As a first negative control, microtubes containing liquid medium and EO were used. Moreover, for each bacterium, a microtube filled with Mueller–Hinton broth containing 5% DMSO and 5 µL of bacterial suspension was used as a second negative control. After incubation, as a measure of bacterial growth, p-iodonitrotetrazolium chloride (INT) 95% (Sigma-Aldrich) was added to all microtubes (growth indicator). The quality of the test was assured by observing no change in dye color in the first negative control microtubes and an obvious reduction of INT color from yellow to pink in the second negative control microtubes (indicating no effect of 5% DMSO on growth). The MIC was the highest sample dilution at which the color change from yellow to pink was undetectable. 

#### 2.7.2. Minimum Bactericidal Concentration Method

The minimum bactericide concentration (MBC) test was conducted by sub-culturing 10 µL from a microtube that did not show a positive result with the MIC test onto a sterile plate containing nutrient agar and incubating the plates overnight at 35–37 °C. The MBC was determined to be the minimum concentration at which no growth occurred in the medium. The chloramphenicol served as the control. Furthermore, the MBC/MIC ratio was estimated in order to recognize the potential mechanism of the tested product. If the value is less than four, the tested compound is bactericidal; if it is equal to or higher than four, the compound is bacteriostatic [38].

### 2.8. In Silico Antibacterial Action Investigation

#### 2.8.1. ADMET In Silico Pharmacokinetics and Drug Similarity Prediction

Using computational technology to identify new drug candidates reduces the number of experimental studies and improves the success rate [39]. For this, we used ADMET (adsorption, distribution, metabolism, excretion, and toxicity) profiling to measure the pharmacokinetic parameters of thymol and ethane to assess their chances of becoming drug candidates in the future.

#### 2.8.2. Molecular Docking

The program Autodock Vina was used to prepare the molecular docking and the chemical structure of thymol [40]. The crystal structure of free BSA with PDB ID: 3VO3 was downloaded from the Protein Data Bank (http://www.rcsb.org, accessed on 15 June 2022). Water molecules were removed and then hydrogen atoms were added to the protein structure. Docking cycles were run using AutoDock Tools version 1.5.6.36. The 3D grid was created using the AUTOGRID37 algorithm to evaluate the interaction energy between the ligand and the protein [41]. The selected ligand was based on flexibility and conformations using the Ascore scoring function, which estimates the free binding energy [42,43,44]. The applied box size was 60 × 60 × 60 Å with a grid resolution of 0.35 Å. The two compounds extracted from cumin essential oils by the aforementioned methods have almost the same activities. Thus, to ensure that the designed molecules are viable drugs, we used the ADMET pharmacokinetic parameters.

## 3. Results and Discussion

### 3.1. Chemical Composition

The chemical composition of the EO of the aerial part of *M. piperita* was analyzed by the GC-MS method. Table 1 describes in detail the chemical compounds of *M. piperita* EO from the two regions studied: Azrou and Ouezzane (Northern Morocco), as well as the properties of each compound, including percentage content (%) and retention time.

In the EO of *M. pepirita* from the Azrou region, a total of 17 compounds were identified. Carvone (70.25%) was the most dominant compound identified, followed by neodihydrocarveol (4.22%), and β-myrcene (4.00%). The minor components were γ-terpinene (2.87%), caryophyllene (2.77%), β-bourbonene (2.26%), α-pinene (1.84%), germacrene D (1.74%), 2-cyclohexen-1-ol (1.66%), 3-Octanol (1.15%), and β-famesene (1.01%). On the other hand, in the EO of *M. pepirita* from Ouezzane, 22 compounds were identified, including a high concentration of sesquiterpenes and monoterpenes, menthol (43.32%) and menthone (29.4%) were the main components in the *M. pepirita,* followed by limonene (7.22%) and isomenthone (6.93%). Minor components were caryophyllene (3.36%), β-pinene (1.41%), pulegone (1.23%), and piperitone (1.21%). In accordance with the results obtained in the present study, a comparison of the *M. pepirita * EO derived from the two regions examined (Azrou and Ouezzane) showed high variability in the content of the main biologically active components. The highest amounts of menthol (43.32%) and menthone (29.4%) were only found in the Ouezzane region, while the highest yield of carvone (70.25%) was found in the Azrou region. In the Ouezzane region, D-carvone was only (0.11%).

Numerous researchers have explored the chemical composition of this plant as well as that of other species. A certain similarity and, at the same time, a great diversity were noted in the phytochemical composition of the various extracts and EO of this ubiquitous species by comparing our results with those of national and international studies.

In the “El Gharb region” of Morocco, GC-MS analysis demonstrated that the primary components of *M. pepirita* EO were menthone (29.24%), levomenthol (38.73%), and eucalyptol (6.75%), which corroborate the findings reported in the present study, with the exception of eucalyptol, which was not found in our screening [45]. Another previous Moroccan work in Taounate pointed out that the chemical composition of peppermint EO analyzed by GPC/MS indicated menthol (46.32%), menthone (7.42%), and 1,8-cinole (6.06%) as the principal components, which is consistent with the data acquired in our case [46]. In a recent investigation, Indian researchers assessed the chemical composition of EOs of *M. piperita* and two other plants, *M. spicata* and *Tagetes minut*. They detected that menthone (29.54%) was considered one of the primary molecules present in *M. piperita,* which is consistent with the Ouezzane region findings, but they also identified other compounds not seen in our data, such as neo-isomenthol (38.64%), neo-menthyl acetate (7.55%), menthofuran (6.49%), and 1,8-cineole (6.31%) [47]. Similarly, in the study by Li et al., menthone was one of the core molecules in peppermint EO and did not show significant changes in its levels when exposed to phenanthrene, as shown in the polycyclic aromatic hydrocarbon model [48]. In addition, Fidan et al. in their recent work aimed to screen the chemical composition of EOs from oils from different species of the genus *Mentha*. The main constituents of the three specimens of *M. piperita* L. were menthol (30.35–41.53%), menthone (17.03–22.00%), 1,8-cineole (4.97–5.87%), menthyl acetate (6.45–10.47%), and iso-menthone (3.67–4.90%) [49].

Furthermore, in another recent study in Spain, menthone (23.1%) was found to be one of the major compounds in *M. piperita* in addition to menthol (47.0%), menthyl acetate (5.2%), menthofurane (3.7%), and neomenthol (3.6%) [50].

On the other hand, the results are completely different from those of the present study regarding the Brazilian EO, which represents (98.0%) of the oil and shows linalool (51.0%), 3-octanol (10.1%), and terpin-4-ol (8.0%) as the main components, with the exception of the carvone compound (23.42%), which is consistent with the results found in the Azrou region as the major compound [51]. Additionally, the peppermint Eos from Colombia were entirely diverged in that the oil had pulegone (44.54%), isomenthol (7.23%), isomenthone (26.15%), and chrysanthone (8.07%) [52]. Using the GC/MS analysis, Tsai et al. in their investigation showed that peppermint EO from Taiwan was rich in menthol (30.35%), menthone (21.12%), trans-carveol (10.99%), and 1, 8-cineole [53]. The oil from Burkina Faso showed menthol (39.3%), menthone (25.2%), menthofuran (6.8%), and menthyl acetate (6.7%) as the principal components [54]. Nonetheless, as described by Desam et al., certain specimens demonstrate markedly different profiles in which monoterpenes hydrocarbon, such as α-terpinene and limonene, or monoterpenes oxygenated such as linalool, pulegone or carvone are prominent candidates [55]. Additionally, our results are different from those obtained in the Iranian study in which α-terpiene (19.7%), pipertitinone oxide (19.3%), trans-carveol (14.5%), and isomenthone (10.3%) were the main molecules. However, menthol (36.02%), menthone (24.56%), menthyl acetate (8.95%), and menthofuran (6.88%) were primarily found in the oil from Saudi Arabia [55]. In another Iranian study, the main ingredients were menthol (45.34%), menthone (16.04%), menthofuran (8.91%), cis-carane (8.70%), 1,8-cineole (4.46%), neo-menthol (4.24%), and limonene (2.22%), as shown by the hydrodistillation method [56]. 

On the other hand, as confirmed by the GS-MS assay, several investigations have shown that carvone (34.94%) was the main volatile molecule identified in peppermint EOs from Colombia (ariel parts) [52], Oman (leaves) [57], Brazil (fresh plant) [51], and India (mint herb) [58], with percentages of 61.53%, 34.94%, 23.42%, and 19.95%, respectively. However, β-Myrcene was found as a minor compound in Oman (0.57%) [57], India (0.10%) [58], Egypt (1.83%) [59], and Serbia (0.11%) [60]. These findings are in agreement with those obtained in the Azrou region (carvone:70.25%; β-Myrcene:4.00%). Among monoterpenes, limonene is one of the major chemical components found in several studies. For example, in Iran (ariel parts) [61], the percentage was 28.8%, in Colombia (ariel parts) [52], Brazil (whole plant) [62], and Pakistan (leaves) [63], the percentages were 12.58%, 37.18%, and 7.58%, respectively, which is consistent with the results identified in the MPEO of Ouezzane.

Generally, according to these previous investigations and our findings, five major chemotypes can be distinguished in this medicinal plant: menthone, menthol, iso-menthone, carvone, and limonene. The variation in the percentages of the main components have been mentioned in several studies. It might be due to the origin of the plant, different environmental factors (geographical, seasonal, and climatic conditions, as well as the effect of sunlight and salinity), hereditary/genetic, nutrition, and chemotype of the plants, which make this complexity among the genus completely expected [55,64]. The impact of different distillation processes on oil yield and composition have been previously documented [65]. The composition and quality of EOs may also be influenced by other factors, such as the crop, time of harvest, or post-harvest handling [66].

### 3.2. Antioxidant Activity

To date, there are several techniques for studying the antioxidant properties of medicinal plants. In the current study, the antioxidant activity of *M. piperita* EOs was assessed through the methods of DPPH, ABTS, H_2_O_2_, and Xanthine assays. Table 2 presents the results of the antioxidant activity of *M. piperita* EOs. From the analysis of EC50 values, the DPPH radical scavenging activity of *M. piperita* EOs from Ouezzane (49.83 ± 0.03 µg/mL) was found to have the highest antioxidant capacity. *M. piperita* EOs from Azrou had the lowest IC_50_ (61.19 ± 0.02 µg/mL) compared to *M. piperita* EOs from Ouezzane. The standard antioxidant ascorbic acid possesses powerful antioxidant activity with an IC_50_ value of 9.03 ± 0.02 µg/mL. The EOs samples exhibited the lowest antioxidant capacity when compared to the standard ascorbic acid. In the ABTS assay, the EOs samples exhibited strong scavenging activity against ABTS radicals. The EOs samples were from different regions with respective IC_50_ values of 124.11 ± 0.01 µg/mL and 111.52 ± 0.05 µg/mL. In the case of the assessment of the ability to reduce the pool of hydroxyl radicals expressed as H_2_O_2_, it was found that the tested EOs from *M. piperita* showed a remarkable effect in terms of antioxidant activity with IC_50_ values of 44.61 ± 0.06 µg/mL and 31.14 ± 0.02 µg/mL, respectively. 

By comparison, the positive control ascorbic acid had hydroxyl radical scavenging activity with an IC_50_ value of 3.23 ± 0.08. The IC_50_ (μg/mL) of *M. piperita* EOs by using a xanthine oxidase assay was found to be 28.74 ± 0.07 μg/mL and 17.49 ± 0.01 μg/mL, while the reference allopurinol was 1.14 ± 0.05 μg/mL. As expected, all two *M. piperita* EOs possessed a powerful antioxidant activity across different antioxidant-based tests. However, there was variation in the antioxidant findings of the two EOs. Plants could be partially attributed to the extraction techniques used, origin, growth stage, and environmental influences [45]. At the same time, our results showed an agreement with previously reported oil and leaf extracts of *M. piperita* from Libya [16]. A study conducted by Sun et al. showed that *M. piperita* EOs from China exhibited remarkable antioxidant properties [67]. Furthermore, our findings were in agreement with studies in which the antioxidant activity could be attributed to the major monoterpenoids such as menthone (29.4%), carvone (70.25%), and Levomenthol (43.32%) [45,68,69]. Several studies have shown that enhanced antioxidant properties are found in EOs [70]. Hence, the chemical compounds of EO from *M. piperita* might work individually or synergistically as natural antioxidants. 

### 3.3. Antidiabetic Activity

One of the strategies adopted to treat diabetes mellitus involves the inhibition of carbohydrate-digesting enzymes such as α-amylase and α-glucosidase which can therefore retard glucose liberation from complex carbohydrates [71].

In this study, both essential oils inhibited the α-amylase activity in vitro, with IC_50_ values of 131.62 ± 0.01 µg/mL and 91.64 ± 0.03 µg/mL, respectively. In the same vein, the capacity of EO from *M. piperita* to inhibit α-glucosidase activity in vitro was evaluated and the result is presented in Table 3. The results revealed that both essential oils exhibited α-glucosidase inhibitory activity with IC_50_ values of 104.32 ± 0.01 µg/mL and 72.41 ± 0.02 µg/mL, respectively.

Acarbose was used as a control positive, which showed α-amylase inhibitory activity with an IC_50_ value of 72.34 ± 0.02 µg/mL and α-glucosidase inhibitory activity with an IC_50_ value of 41.17 ±0.01 µg/mL. Moreover, the same study reported that these EOs also have a remarkable inhibition action against pancreatic lipase activity with IC_50_ values of 86.24 ± 0.01 µg/mL and 59.32 ± 0.08 µg/mL, respectively, whereas that of orlistat (a standard reference) was 16.08 ± 0.01 µg/mL. 

Regarding *Mentha* species, a study conducted by Bouyahya et al. showed that the essential oils of *Mentha viridis* exhibited a potent inhibition against α-Amylase (IC_50_ = 101.72 ± 1.86 μg/mL) and α-Glucosidase (IC_50_ = 86.93 ± 2.43 μg/mL) [13]. In other studies, *Mentha canalenisa* [72] and *Mentha longifolia* [73] showed an important inhibition against α-amylase and α-glucosidase activities. The promising results of these two species could be attributed to their chemical compounds, which are known for their antidiabetic potential [74].

### 3.4. Anti-Inflammatory Activity

#### 3.4.1. In Vivo Anti-Inflammatory Activity

It is now well established that inflammation is considered an essential baseline response, leading to the development of many chronic diseases, including cancer, diabetes, obesity, sepsis, and atherosclerosis. Indeed, the risk of these disorders is significantly increased by the presence of chronic inflammation, suggesting that suppression of this inflammation may be an attractive approach for the prevention and therapy of these conditions, particularly cancer [75,76]. To this end, we examined the anti-inflammatory properties of the *M. pepirita* EOs we identified. Details of the effectiveness of *M. pepirita* EOs on carrageenan-induced edema are presented in Table 4 and Table 5.

The EOs of *M. pepirita* from the Ouezzane and Azrou areas demonstrated remarkable (*p* < 0.05) anti-inflammatory potency compared to the control and standard groups (Table 5). At 1 h and 30 min, the Ouezzane EOM extract and the Azrou EOM presented an edema inhibition of 49.69% and 32.09%, respectively, compared to the non-steroidal anti-inflammatory drug standard Indomethacin at 69.77%. Nevertheless, at three hours, the EO of *M. pepirita* from the Ouezzane and Azrou regions showed a higher level of inhibition with 58.02% and 38.69%, respectively, compared to the reference drug Indomethacin with 70.23% for the same period (Table 5).

Our evidence appears to be similar to previous studies demonstrating the anti-inflammatory activity of *M. piperita* extracts and EO [77,78,79]. For example, Sun et al. found that volatile EO and aqueous extract of Chinese-grown peppermint were able to suppress the edematous response in a croton oil-induced mouse ear edema model by downregulating nitric oxide (NO) and prostaglandin E2 (PGE2) generation [67]. 

Furthermore, there was a remarkably strong anti-inflammatory benefit in the groups receiving *M. piperita* EO cultivated in Romania (500 mg/kg), 4 h following the initiation of the inflammation, as shown in the in vivo study conducted by Mogosan, C et al. [80]. From another study carried out by Kehili, S, and collaborators, Algerian peppermint EO could be considered a promising anti-inflammatory drug. It was able to reduce acute ear edema by 38.09% and 36.50% after topical application at doses of 200 and 20 µL/kg, respectively [77]. In fact, the development of edema in the rat’s hind paw following carrageenan administration could be distinguished as a two-step event. In the initial phase, induction of the inflammatory response to carrageenan (0–1 h) is driven by the liberation of bradykinin, histamine, reactive oxygen species (ROS), complement, and serotonin. Subsequently, to accelerate the swelling phase (2–4 h), an enhanced release of PGE in the inflammatory zone has been proven. It has also been suggested that the activation of toll-like receptor (TLR) 2/6 and TLR4/6 are the main pathways by which the inflammatory responses of carrageenans are induced [81].

#### 3.4.2. In Vitro Anti-Inflammatory Activity

Inflammation is an essential part of life and is normally helpful as well as beneficial for health. However, inflammatory responses can also lead to overreaction and damaging consequences. The signs of inflammation are mediated by inflammatory mediators, namely the invasion of leukocytes and the escape of small vessel plasma into the inflamed tissue [82]. As lipoxygenase (LOX) products of arachidonate metabolism have been implicated in inflammatory processes [83]. 5-LOX is primarily known for its key role in the generation of leukotrienes in diseases and health that serve as pro-inflammatory mediators, formed from arachidonic acid. Nevertheless, 5-LOX also plays a major function in lipid mediator biosynthesis, which is anti-inflammatory and/or promotes the rescue of inflammation-like lipoxins [84].

LOX inhibition is the most common method used to investigate the anti-inflammatory effect in vitro. In the present study, the potency of *M. pepirita* EO was evaluated for its anti-inflammatory effect via the 5-LOX inhibition test. The findings of the 5-LOX inhibitory activity of the tested EOM from Ouezzane and EOM from the Azrou region were provided as outlined in Table 6, indicating a significant activity of EOM from Ouezzane with an IC_50_ equal to 11.64 ± 0.02 µg/mL better than the IC_50_ of the Azrou EO which is equal to 23.84 ± 0.03 µg/mL. Our data from both regions showed lower IC_50_ values when compared to the positive control quercetin, which had a value of 3.51 ± 0.01 µg/mL. These results were supported by several investigations which demonstrated the potent anti-inflammatory property of this plant in vitro [77,85,86,87]. Therefore, a variety of mint EOs may possess anti-inflammatory characteristics. However, further in vivo and in vitro studies are necessary to ensure efficacy and safety.

### 3.5. Antibacterial Activity

**Figure 1:**
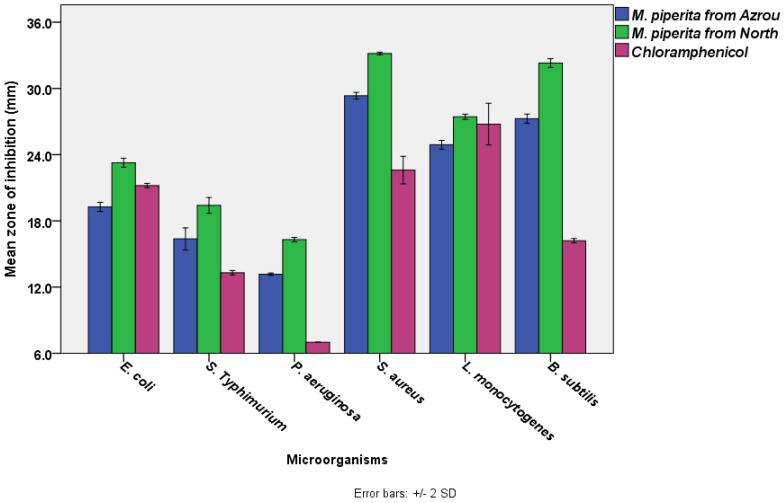
The antimicrobial activities of *M. piperita* EOs from the Azrou and Ouezzane regions using disc diffusion test (The diameter of the paper disc is 6.0 mm, the concentration of *M. piperita* from both regions was 100% *v*/*v*. The concentration of chloramphenicol was 30 µg /mL).

The results showed that *M. piperita* EOs from the Azrou and Ouezzane areas had significant antibacterial activity (ANOVA, *p* < 0.05). According to the disc diffusion test (Figure 1), the most susceptible bacterium to *M. piperita* EO from the Azrou region was *S. aureus* (29.3 ± 0.2 mm), followed by *B. subtilis* (27.3 ± 0.2 mm), *L. monocytogenes* (24.9 ± 0.2 mm), *E. coil* (19.3 ± 0.2 mm), and *S. typhimurium* (16.4 ± 0.5 mm), respectively. Conversely, the least susceptible bacterium to that EO was *P. aeruginosa* (13.2 ± 0.1 mm). Similarly, but with a higher degree of activity, the bacterium most sensitive to *M. piperita* EO from the north area was *S. aureus* (33.2 ± 0.1 mm), *B. subtilis* (32.3 ± 0.2 mm), *L. monocytogenes* (27.4 ± 0.1 mm), *E. coil* (23.3 ± 0.2 mm), and *S. typhimurium* (19.4 ± 0.4 mm), respectively. *P. aeruginosa* was the least sensitive bacteria to that EO (16.3 ± 0.1 mm). 

Statistical analysis (a Paired Samples T-test) indicated that *M. piperita* EOs from the Ouezzane region had significantly higher antibacterial activity than M. piperita EOs from the Azrou region. *S. aureus* exhibited the greatest zone inhibition, which was followed by *B. subtilis*, *L. monocytogenes*, *E. coli*, *S. typhimurium*, and *P. aeruginosa*. It is obvious that gram-positive bacteria (ranging from 33.2 ± 0.1 to 24.9 ± 0.2 mm) were more susceptible than gram-negative bacteria (ranging from 23.3 ± 0.2 to 13.2 ± 0.1 mm). Additionally, the essential oils exceeded the standard antibiotic in terms of effectiveness. In general, our results are in agreement with previous studies which reported that *Mentha piperita* essential oils from Morocco (harvested in gardens) have substantial antibacterial activity against various bacterial strains [46,88]. *M. piperita* also showed noticeable antibacterial activity against emerging multi-drug-resistant bacteria [89], and its efficacy was reported to be comparable to the standard antibiotics used in the disc diffusion assay [16], which supports our current findings (Figure 1). The standard antibiotic, chloramphenicol has a wide range of action against both gram-positive and gram-negative bacteria as well as Rickettsia. It works by attaching to ribosomes and inhibiting the production of bacterial proteins [90]. Our results also showed that variations in locations could influence the antibacterial efficacy of *M. Piperita*. This was also observed by other researchers, who cited that the geographical climate, the type of soil, the season, and the processing technology all have a direct influence on the phytochemical contents of plants and, in turn, their antibacterial activity [91,92].

On the other hand, the results of MIC and MBC supported the findings of the disc diffusion test. As shown in Table 7, based on the MIC ratios, the lowest concentration of *M. piperita* EO from the Azrou area that inhibits the visible bacterial growth ranged from 0.05 to 3.12 mg/mL; whereas the lowest concentration of *M. piperita* EO from Ouezzane that inhibits the obvious bacterial growth ranged between 0.05 to 6.25 mg/mL. Here, it is evident that the antibacterial properties of *M. piperita* EO from Ouezzane are more effective than *M. piperita* EO from the Azrou region. Similarly, according to the MBC values, the lowest concentration of the essential oils that kills test bacteria (in vitro), were mostly similar to the MIC values, except with *P. aeruginosa* (12.5 mg/mL) and *E. coli* (1.56 mg/mL) for *M. piperita* from the Azrou region, and *P. aeruginosa* (25.0 mg/mL), *S. typhimurium* (3.12 mg/mL), *B. subtilis* (0.1 mg/mL), and *L. monocytogenes* (0.2 mg/mL), for *M. piperita* from the Ouezzane area. 

Thus, our data on MIC and MBC is consistent with previously published reports, indicating that these plants’ essential oils are necessary to consider using as potent antibacterial agents [16,93], including human antibiotic-resistant bacteria [94] and food-spoiled bacteria [95]. The MBC/MIC ratio gives a general picture of the nature mechanism of the antibacterial agent (Table 8), which ranged from 1.0 to 2.0 for *M. piperita* from both regions, indicating that *M. piperita* from the two locations has a substantial bactericidal activity. This is based on the criterion that the plant’s antibacterial agent is termed bactericidal when the MBC/MIC ratio is less than 4.0 and bacteriostatic when it is more than 4.0 [96,97].

In general, according to the disc diffusion, MIC, and MBC results, *M. piperita* EO from both regions has noticeable antibacterial activity against the tested bacteria. The variation between the antibacterial degrees was attributed to the phytochemical constituents, which play an intrinsic role in the antibacterial potential of medicinal plants [98,99]. There are 25–30 species in the genus *Mentha* [100], most of which have antibacterial properties, and they are found in moderate areas of North and South Africa, Eurasia, and Australia [101]. The current study has also proven this plant’s traditional usage in Morocco as a remedy for some microbial-based diseases. It is reported that the ethnopharmacological survey showed that Moroccans consume *Mentha* daily in tea infusions and traditionally use it to treat gastrointestinal disorders, respiratory diseases, endocrine disorders, fevers, diabetes, migraines, hypertension, cardiac diseases, rheumatism, pains, nervous disorders, obesity, musculoskeletal ailments, urinary diseases, and skin infections [102].

In sum, *M. piperita* essential oil is a powerful antibacterial agent and it may be used as a natural drug to treat a range of contagious diseases caused by bacterial pathogens. Additional future studies on the mode of action of the essential oils of *M. piperita* in vitro and in vivo, as well as other pharmacological and toxicological studies, are recommended.

### 3.6. Molecular Docking 

The pkCSM online tool was used to report ADMET in silico properties (Table 9). ADMET properties and drug acceptability were predicted using the online tool SwissADME. An absorbance value below 30% indicates low absorbance, and all compounds proved an absorbance value above 90%, indicating good absorbance in the human gut. The water diffusion value (VDss) is considered high if it is greater than 0.45. In addition, blood-brain barrier (BBB) permeability for a given compound, log BB > 0.3, means that the drug readily crosses the blood-brain barrier f, whereas log BB < −0.1 indicates that the drug is poorly distributed in the brain.

Standard values for central nervous system (CNS) permeability with log PS > −2 mean that it can enter the CNS, while log PS < −3 means that it is difficult for the drug to enter the CNS [103,104,105].

The results indicated that both compounds had significant potential to cross significant barriers. The metabolic rate indicates the chemical biotransformation of a drug by the body. Therefore, drugs produce several metabolites which may have different physicochemical and pharmacological properties. It is necessary to take into account the metabolism of drugs and inter-drug reactions [106,107]. The results indicated that both compounds had significant potential to cross barriers. The metabolic rate indicates the chemical biotransformation of a drug by the body [108,109]. Therefore, drugs produce several metabolites, which may have different physicochemical and pharmacological properties [110,111].

In contrast, inhibition of the cytochrome CYP3A4 was the most important phenomenon in this study. The two selected compounds were found to be the substrate of CYP3A4 and the inhibitor of CYP3A4. Clearance is a constant that describes the relationship between the concentration of the drug in the body and the rate of drug elimination. Therefore, a low total clearance value means a higher persistence of the drug in the body, and thymol has a good persistence of the drug in the body. Fortunately, all the compounds we have designed are non-toxic. In conclusion, both have good pharmacokinetic properties.

**Figure 2:**
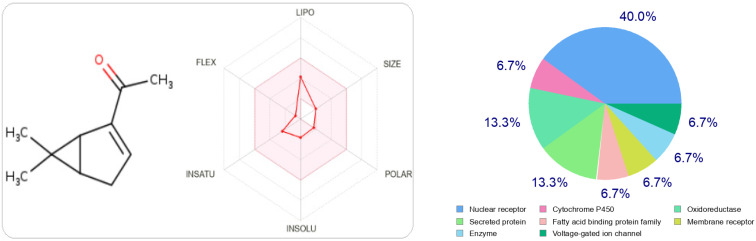
The radar of bioavailability allowing a drug resemblance of carvacrol and the probability percentages attached to each type of bioactive target.

**Figure 3:**
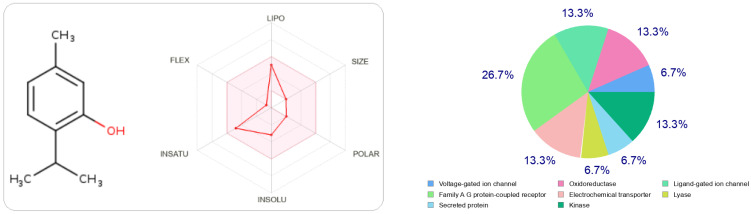
The radar of bioavailability allowing a drug resemblance of thymol and the probability percentages attached to each type of bioactive target.

In Figure 2 and Figure 3, we can see that both molecules represent the optimal range for each property (lipophilicity: XLOGP3 between −0.7 and +5.0, size: MW between 150 and 500 g/mol, polarity: TPSA between 20 and 130 Å^2^, solubility: log S not greater than six, saturation: fraction of carbons in sp 3 hybridization not less than 0.25, and flexibility, no more than nine rotational bonds. In this example, both compounds should be orally bioavailable as they are not too flexible and not too polar.

At the same time, if we make a comparison of the targets attracted by each ligand, either thymol or carvacrol, we conclude that they have different regions.

In order to confirm that thymol is the best compared to carvacrol, we performed docking for thymol. 

The type most likely to be attracted to thymol is the ligand-gated ion channel at 26.7%, followed by oxidoreductase, electrochemical transporter, and kinase participating at the same probability rate, 13.3%. On the other hand, carvacrol has other preferred regions, nuclear receptors (40%), followed by oxidoreductases (13.3%) and secreted proteins, which have the same probability value as oxidoreductases.

**Figure 4:**
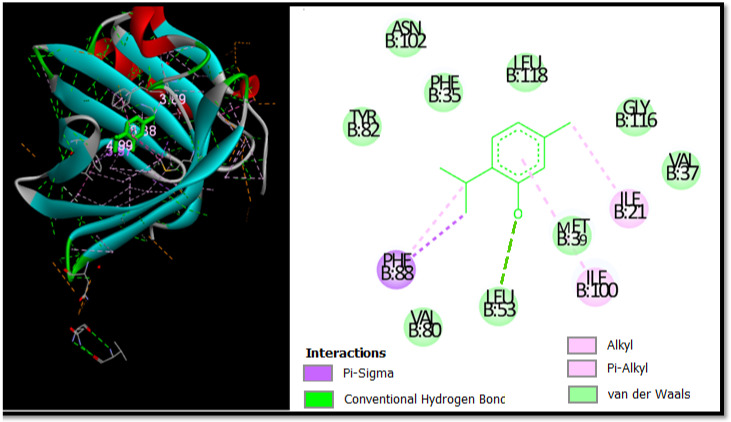
The 2D and 3D docking poses proving thymol interactions in binding sites.

In this study, Autodock software was used to determine the binding site of thymol on BSA. Thymol was docked to BSA to prove the preferred binding sites. Figure 4 shows the best conformation of the binding mode between thymol and BSA. It has been reported that the binding sites of different exogenous or endogenous ligands on BSA are located in subdomains IIA and IIIA, referred to as Sudlow I and II sites, respectively, and the drug binding sites located in these subdomains [112].

In contrast, in the current study, the docking results proved that thymol binds in the binding pocket of subdomain IA. This is not a common place for thymol to bind, but it was recently found to be a place where chemicals could bind to albumin [113]. 

Figure 4 is used to further prove the docking results of the thymol-BSA complex. Thymol forms H-bonds with the residue Leu53 (3.69 Å). In addition, thymol formed alkyl and pi-alkyl bonds with Ile-21 Ile-100, as well as two pi-Sigma bonds with Phe-88. It can be seen that the majority of the interactions are van der Waals and the residues involved in this type are Phe-35, Leu-118, Val-80, Ala-26, Leu-53, Asn-102, Tyr-82, Val-37, and Gly-116 with bonding energy of −9.48 kcal mol^−1^.

In summary, this theoretical step is used to predict the activity or target attracted by these two molecules, such as thymol and carvacrol, either by ADMET or molecular docking methods. We can confirm that these two molecules have significant activity, and this is interpreted as the appropriate experimental part.

## 4. Conclusions

Essential oil of *M. piperita* from the two different geographical areas revealed that there was a difference in its phytochemical characteristics according to the region, and this variation in its chemical properties yielded distinct effectiveness in the biological properties. The collected plants from both locations exhibited very attractive anti-inflammatory potency, indicating that the primary compounds in *M. piperita* EOs could potentially have significant applications as anti-inflammatory drugs in the future. Nevertheless, investigations in animal models with chronic inflammation could yield very intriguing insights into the mechanisms by which these major compounds act against inflammation. Furthermore, a possible study of the antimicrobial activity of these essential oils could be conducted using antibiotic-resistant pathogens and bacteria inducing a chronic inflammatory response. This might provide very valuable findings regarding these main components as natural antibiotics. In addition, the majority of current investigations are focused on the MPEO mixture, with a limited number of studies on menthol. Given that in our context MPEO is a mixture of several components, it is appropriate to draw more attention to the other constituents of MPEO, e.g., carvone, Levomenthol, and Menthone, which will greatly contribute to the understanding of the full mechanisms of action of MPEO in the foreseeable future.

## Figures and Tables

**Table 1:** Chemical composition of *M. pepirita* EO.

Compounds	R_t_	KI	%EO Azrou	% EO Ouezzane	Formula
α-Thujene	2.014	918	0.04	0.02	C_10_H_16_
α-Pinene	2.081	936	1.84	0.88	C_10_H_16_
Camphene	2.250	938	0.37	0.06	C_10_H_16_
β-Pinene	2.735	978	--	1.41	C_10_H_16_
β-Myrcene	3.174	981	4.00	0.12	C_10_H_16_
α-Terpinene	3.558	1011	--	0.06	C_10_H_16_
Limonene	3.907	1015	--	7.22	C_10_H_16_
(Z)-β-Ocimene	4.245	1027	0.39	--	C_10_H_16_
γ-Terpinene	4.493	1052	2.87	0.1	C_10_H_16_
Menthone	7.118	1152	--	29.4	C_10_H_18_O
Isoborneol	7.321	1159	1.72	--	C_10_H_18_O
Menthol	8.133	1172	--	43.32	C_10_H_20_O
α-Terpineol	8.257	1176	--	0.83	C_10_H_18_O
Pulegone	9.057	1229	--	1.23	C_10_H_16_O
Carvone	9.169	1234	70.25	0.11	C_10_H_14_O
Piperitone	9.428	1244	--	1.21	C_10_H_16_O
Isomenthone	10.679	1288	--	6.93	C_10_H_18_O
Neodihydrocarveol	11.693		4.22	--	C_10_H_18_O
β-Bourbonene	12.719	1378	2.26	0.42	C_15_H_24_
Caryophyllene	13.756	1423	2.77	3.36	C_15_H_24_
α-Humulene	14.409	1448	0.48	0.12	C_15_H_24_
epi-Bicyclosesquiphellandrene	14.646	1467	0.73	--	C_15_H_24_
β-Famesene	14.939	1474	1.01	0.07	C_15_H_24_
Germacrene D	14.950	1479	1.74	0.09	C_15_H_24_
γ-Elemene	15.243		0.41	--	C_15_H_24_
α-Muurolene	15.930	1496	0.12	0.22	C_15_H_24_
Caryophyllene oxide	16.449		--	0.26	C_15_H_24_O
	Total identified compounds%		95.22	97.44	
	Monoterpene hydrocarbons		9.51	9.87	
	Oxygenated monoterpenes		76.19	83.03	
	Sesquiterpenes hydrocarbons		9.52	4.28	
	Oxygenated sesquiterpenes		--	0.26	

**Table 2:** Antioxidant effect of *Mentha piperita* EOs.

EOs IC_50_ (µg/mL)	DPPH	ABTS Test	Xanthine Oxidase	H_2_O_2_
MPEO of Azrou	61.19 ± 0.02 ^c^	124.11 ± 0.01 ^c^	28.74 ± 0.07 ^c^	44.61 ± 0.06 ^c^
MPEO of Ouezzane	49.83 ± 0.03 ^b^	111.52 ± 0.05 ^b^	17.49 ± 0.01 ^b^	31.14 ± 0.02 ^b^
Ascorbic acid	09.03 ± 0.02 ^a^	74.81 ± 0.01 ^a^	--	3.23 ± 0.08 ^a^
Allopurinol	--	--	1.14 ± 0.05 ^a^	--

Different superscript letters in the same column indicate a significant difference (*p* < 0.05).

**Table 3:** Antidiabetic effect of *Mentha piperita* EOs.

EOs IC_50_ (µg/mL)	α-Amylase	α-Glucosidase	Lipase
MPEO *from Azrou*	131.62 ± 0.01 ^c^	104.32 ± 0.01 ^c^	86.24 ± 0.01 ^c^
MPEO from Ouezzane	91.64 ± 0.03 ^b^	72.41 ± 0.02^b^	59.32 ± 0.08 ^b^
Acarbose	72.34 ± 0.02 ^a^	41.17 ± 0.01 ^a^	--
Orlistat	--	--	16.08 ± 0.01 ^a^

Different superscript letters in the same column indicate a significant difference (*p* < 0.05).

**Table 4:** Effect of *M. pepirita* essential oils on carrageenan-induced rat paw edema.

Treatment Group	Mean Edema Volume (Left-Right Paw) mL		
	1 h 30 min	3 h	6 h
Control	0.483 ± 0.02	0.598 ± 0.04	0.473 ± 0.07
Indomethacin	0.146 ± 0.04 *	0.178 ± 0.01 *	0.191 ± 0.01 *
MPEO of Ouezzane	0.243 ± 0.05 *	0.251 ± 0.014 *	0.233 ± 0.02 *
MPEO of Azrou	0.328 ± 0.01 *	0.367 ± 0.02 *	0.358 ± 0.03 *

* shows the significant difference (*p* < 0.05).

**Table 5:** Percentage of inflammation inhibition by *M. pepirita* on carrageenan-induced rat paw edema.

Treatment Group	Percentage Inhibition of Edema (%)		
	1 h 30 min	3 h	6 h
Indomethacin	69.77	70.23	59.61
MPEO of Ouezzane	49.69 *	58.02 *	50.73 *
MPEO of Azrou	32.09 *	38.69 *	24.31 *

* shows the significant difference (*p* < 0.05).

**Table 6:** In vitro anti-inflammatory.

Assay (IC_50_ μg/mL)	MPEO of Ouezzane	MPEO of Azrou	Quercetin
5-Lipoxygenase	11.64 ± 0.02 ^b^	23.84 ± 0.03 ^c^	3.51 ± 0.01 ^a^

Different superscript letters in the same column indicate a significant difference (*p* < 0.05).

**Table 7:** The MIC, MBC, and MBC/MIC values of *M. piperita* EOs from the Azrou and Ouezzane regions.

Bacteria	*M. piperita* from Azrou (100% *v*/*v*)			*M. piperita* from Ouezzane (100% *v*/*v*)			Chloramphenicol (30 µg /mL)		
	MIC	MBC	MBC/MIC	MIC	MBC	MBC/MIC	MIC	MBC	MBC/MIC
*E. coli* ATCC 25922	0.39	0.39	1.0	0.78	0.78	1.0	8.0	8.0	1.0
*S. typhimurium* ATCC 700408	0.78	1.56	2.0	1.56	3.12	2.0	64.0	64.0	1.0
*P. aeruginosa* ATCC 27853	3.12	12.5	4.0	6.25	25.0	4.0	>64.0	>64.0	1.0
*S. aureus* ATCC 29213	0.05	0.05	1.0	0.05	0.05	1.0	4.0	4.0	1.0
*L. monocytogenes* ATCC 13932	0.1	0.1	1.0	0.1	0.2	2.0	2.0	2.0	1.0
*B. subtilis* ATCC 6633	0.05	0.05	1.0	0.05	0.1	2.0	32.0	32.0	1.0

**Table 8:** ADMET in silico prediction of thymol and carvacrol.

Compounds	Absorption	Distribution			Metabolism							Excretion	Toxicity
	Intestinal absorption (human)	VDss (human)	BBB permeability	CNS permeability	Substrate		Inhibitor					Total clearance	AMES toxicity
					CYP								
					2D6	3A4	1A2	2C19	2C9	2D6	3A4		
	Numeric (%absorbed)	Numeric (log L/kg)	Numeric (log BB)	Numeric (log PS)	Categorical (yes/no)							Numeric (log ml/min/kg)	Categorical (yes/no)
Carvacrol	97.834	0.515	0.676	−2.423	No	No	No	No	No	No	No	−0.004	No
Thymol	90.843	0.512	0.407	−1.664	No	No	Yes	No	No	No	yes	0.211	No

**Table 9:** Drug-likeness prediction and Medicinal Chemistry of thymol and carvacrol.

Compounds	Drug-Likeness					Medicinal Chemistry		
	Lipinski	Ghose	Veber	Egan	Muegge	PAINS	Synthetic Accessibility	Leadlikeness
Carvacrol	Yes	No	Yes	Yes	No	0 alert	3.67	No
Thymol	Yes	no	yes	yes	No	0 alert	1.00	No

## Data Availability

The data used to support the findings of this study are included within the article.
